# Acute thrombosis of bioprosthetic mitral valve

**DOI:** 10.1186/1749-8090-8-185

**Published:** 2013-08-27

**Authors:** Jin-Tae Kwon, Tae-Eun Jung, Dong-Hyup Lee

**Affiliations:** 1 Department of Thoracic and Cardiovascular Surgery, College of Medicine, Yeungnam University, Daemyeong 5-dong, Nam-gu, Daegu 705-717, Korea

**Keywords:** Mitral valve replacement, Bioprosthetic valve, Thrombosis

## Abstract

We report a case of acute thrombosis of bioprosthetic mitral valve in a 59 year–old Korean female, who underwent a mitral valve replacement with a 25 mm Carpentier - Edwards PERIMOUNT Plus bioprosthesis (Edwards Lifesciences, Inc.; Irvine, CA, USA) and a mini-Maze procedure for correction of mitral stenosis (MS) and atrial fibrillation (AF). On the 10th postoperative day, the patient began to complain of increasing dyspnea and general malaise. Her symptoms worsened and developed into pulmonary edema. Echocardiography revealed a mean diastolic pressure gradient (MDPG) of 10 mmHg across the mitral valve and pressure-half time (PHT) of 166 msec. Due to progressive decompensated heart failure, the patient underwent a repeat sternotomy to replace the bioprosthetic mitral valve. Intraoperatively, we found a thrombosis around the bioprosthetic mitral valve. We excised the bioprosthetic mitral valve and replaced it with a 27 mm ATS mechanical valve (ATS medical, Inc.; Minneapolis, MN, USA). We experienced a rare case that required an early reoperation for a thrombosis of the bioprosthetic valve.

## Background

The advantage of using a bioprosthetic valve over a mechanical valve is that it lowers the incidence of thrombosis, avoiding the need for anticoagulant medication in the long term.

The reported incidence of bioprosthetic valve thrombosis on routine echocardiography surveillance is approximately 6% [1]. However, the occurrence of acute thrombosis of the bioprosthetic mitral valves during the early postoperative period is rare.

We experienced a rare case requiring an early reoperation for a thrombosis of the bioprosthetic valve.

## Case presentation

A 59-year old Korean female was referred with shortness of breath. She was diagnosed with moderate mitral stenosis (MS, valve area: 1.07 cm^2^ by 2D) and atrial fibrillation (AF). Preoperative echocardiography showed a large left atrium (LA Volume Index: 81 ml / m^2^).

She underwent preservation of the sub-valvular apparatus of posterior leaflet of the mitral valve and replacement with a 25 mm Carpentier – Edwards PERIMOUNT Plus bioprosthesis (Edwards Lifesciences, Inc.; Irvine, CA, USA) and a mini-Maze procedure. Due to her history of hemorrhagic stroke, we selected the bioprosthetic valve in order to avoid anticoagulation. Her body surface area (BSA) was 1.72 m^2^. The intraoperative transesophageal echocardiogram after the first valve replacement shows no evidence of leaflet entrapment, and bypass weaning was uneventful.

**Figure 1:**
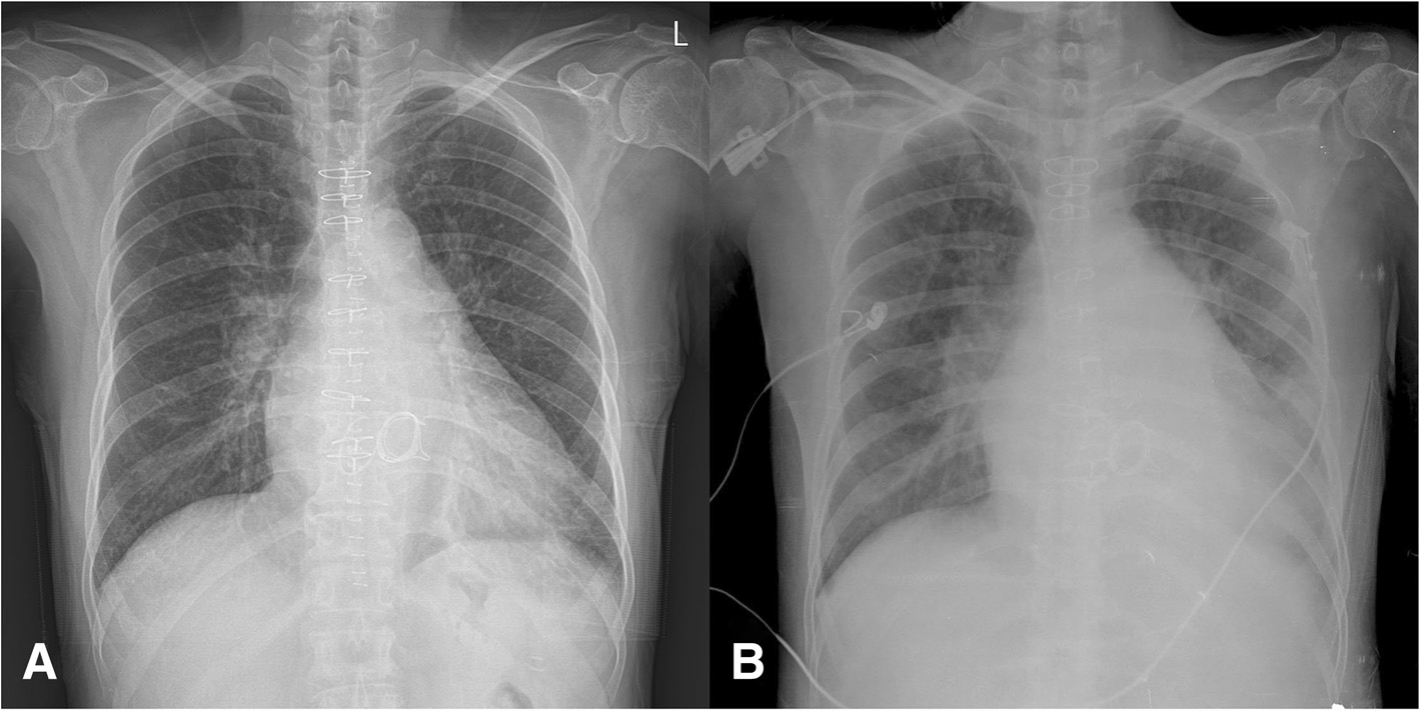
**Chest x-ray.** Postoperative sixth day chest x-ray **(A)**. On the postoperative 12th day, chest x-ray showed interstitial edema **(B)**.

**Figure 2:**
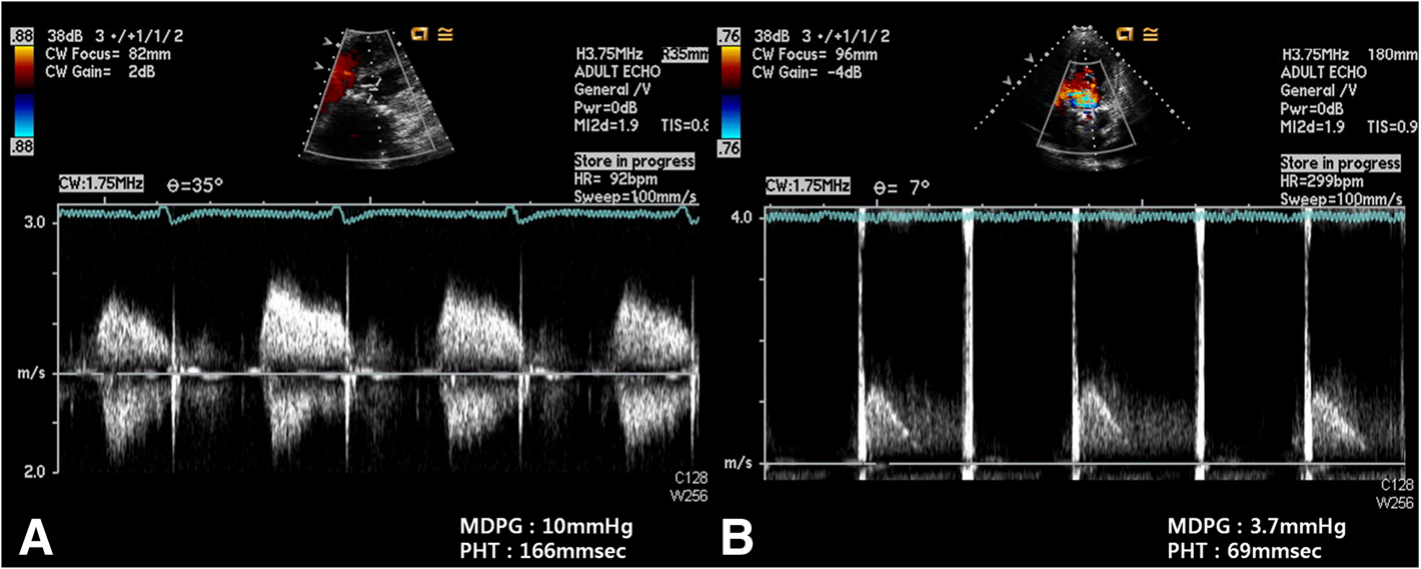
**Transthoracic echocardiogram.** An increased mean diastolic pressure gradient of 10 mmHg after BVR **(A)**. Following reoperation, a comparable transthoracic image depicting normalization of mean gradient **(B)**.

A post operative electrocardiogram showed sinus rhythm. On the first postoperative day, the patient was started on an anticoagulant with Coumadin, and an international normalized ratio (INR) of greater than 2 was reached on the sixth postoperative day. Recovery was uneventful and on the second postoperative day, the patient was transferred to the ward. After a transfer to the general ward, the patient complained of mild dyspnea on exertion; however, sinus rhythm was still present on an electrocardiogram. On the 10th postoperative day, the patient began to complain of increasing dyspnea and general malaise. During the next night, her symptoms worsened and developed into a left side pleural effusion and interstitial edema (Figure 1). A transthoracic echocardiography detected a mean diastolic pressure gradient (MDPG) of 10 mmHg across the mitral valve, but no thrombus in LA. The calculated pressure-half time (PHT) was 166 msec (Figure 2). Clinically, evidence of MS was observed. There was no underlying coagulation disorder. The ejection fraction decreased from 57% to 46%, compared to the preoperative ejection fraction.

**Figure 3:**
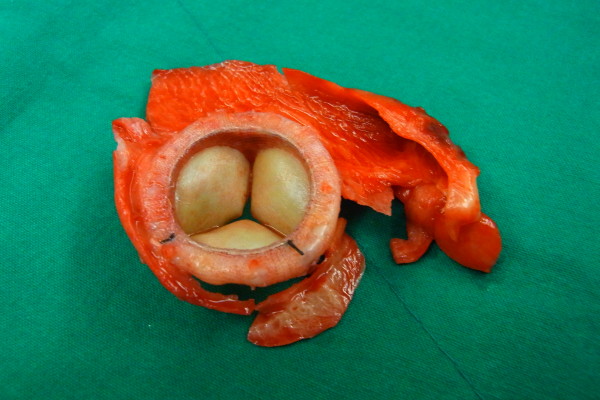
Thrombus is visible on the bioprosthetic valve.

The patient was started on intravenous diuretics and inotropics. However, due to progressive decompensated heart failure, a repeat sternotomy was performed on the 14th postoperative day. Intraoperatively, we observed a thrombus on the bioprosthetic valve and on the posterior left atrial wall (Figure 3). In addition, we noticed that the motion of the valve leaflet was slightly restricted. The bioprosthetic valve was removed and replaced with a 27 mm ATS mechanical valve (ATS Medical, Inc.; Minneapolis, MN, USA). Postoperatively, she showed an improvement of symptoms, and repeat echocardiography showed a MDPG of 3.7 mmHg, PHT of 69 msec, a well functioning prosthetic valve, and no sign of thrombosis. She was discharged on Coumadin with an INR target of 2 to 2.5.

## Discussion

A few studies on the diagnostic characteristics and management of late bioprosthetic valve thrombosis have been reported.

Patients with bioprosthetic aortic or mitral valve replacement have high risk of thromboembolism during the first 10 days [2]. However, within two weeks postoperatively, the occurrence of a thrombosis in bioprosthetic mitral valves is very rare.

Some precipitating factors of the thrombotic process include acting as an underlying coagulopathy, left ventricular dysfunction, low cardiac output, atrial fibrillation, large LA size, and prior history of thromboembolic events [3].

A few cases involving the preservation of the mitral valve apparatus as a predisposing factor for bioprosthetic valve thrombosis have been reported because a complete preservation of the mitral valve apparatus during mitral replacement may lead to an increased incidence of early bioprosthetic thrombosis, although such incidences appear to be rare [4]. However, a complication of mitral valve replacement, such as a left ventricular rupture could be prevented by maintaining the tethering effect of the intact subvalvular apparatus [5]. In addition, there are isolated reports of bioprosthetic thrombosis after a complete valve excision [3].

Incidence of thrombo-embolism associated with AF ablation is reported to be between 0% and 7%; therefore, the 2007 Heart Rhythm Society expert consensus statement recommends a minimum period of two months of warfarin anticoagulation [6]. Although the policy of postoperative anticoagulation are different in each heart center, it took a long time for INR to reach 2.0 on the 6th postoperative day. We should have taken care of more optimal anticoagulation. The American College of Cardiology/American Heart Association (ACC/AHA) guidelines recommend an administration of warfarin for three months, following bioprosthetic valve replacement (BVR) [7]. The risk is particularly high in the first few days after surgery, and many centers start UFH as soon as the risk of increased surgical bleeding is reduced (usually within 24 to 48 h) with maintenance of aPPT between 55 and 70 seconds. After an overlap of UFH and warfarin for 3 to 5 days, UFH may be discontinued when INR reaches 2.0 to 3.0 [7].

In our case, the patient had a large LA size, and underwent posterior leaflet preservation and a mini-Maze procedure. In this case, even though multiple factors are contributed to acute bioprosthetic thrombosis, we should keep up with current anticoagulation guidelines following the BVR and radioablative Maze procedure.

Further studies are needed in order to clarify the anticoagulation strategy after BVR.

Through this case, we also realized the importance of postoperative echocardiographic surveillance in high risk groups.

## Conclusions

The occurrence of bioprosthetic valve thrombosis is uncommon. However, there are some predisposing factors of bioprosthetic valve thrombosis; these include underlying coagulopathy, left ventricle dysfunction, AF, large LA size, prosthetic mismatch, and radioablative Maze procedure.

Therefore, an anticoagulant medication should be maintained and echocardiographic surveillance should be considered for patients in high risk groups.

### Consent

Written informed consent was obtained from the patient for publication of this case report and accompanying images. A copy of the written consent is available for review by the Editor-in-Chief of this journal.

## Competing interests

The authors declare that they have no competing interests.

## Authors’ contributions

JK and TJ wrote the draft of the manuscript and obtained the written consent. DL performed the literature review and participated in the manuscript writing and helped to the final writing of the paper and gave final approval of the manuscript. All authors have read and approved the final manuscript.
